# 2-[(2-Hy­droxy-4-meth­oxy­benzyl­idene)aza­nium­yl]benzoate monohydrate

**DOI:** 10.1107/S1600536810023949

**Published:** 2010-06-26

**Authors:** Zhi-Xi Hang, Bo Dong, Xing-Wen Wang

**Affiliations:** a College of Biological and Chemical Engineering, Anhui Polytechnic University, Wuhu 241000, People’s Republic of China; b College of Chemical Engineering, Nanjing Forestry University, Nanjing 210037, People’s Republic of China; c Department of Biological and Enviromental Engineering, Hefei University, Hefei 230022, People’s Republic of China

## Abstract

In the title compound, C_15_H_13_NO_4_·H_2_O, the Schiff base exists in a zwitterionic form and a bifurcated intra­molecular N—H⋯(O,O) hydrogen bond generates two *S*(6) rings. The dihedral angle between the two benzene rings is 25.8 (2)°. The crystal structure is stabilized by inter­molecular O—H⋯O hydrogen bonds.

## Related literature

For a related compound and background references to Schiff bases, see: Hang (2010 ▶). For related structures, see: Alpaslan *et al.* (2010*a* ▶,*b* ▶); Aritake *et al.* (2010 ▶); Bahron *et al.* (2010 ▶).
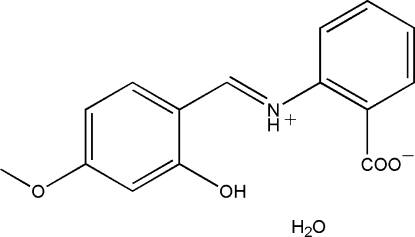


## Experimental

### 

#### Crystal data

C_15_H_13_NO_4_·H_2_O
*M* *_r_* = 289.28
Triclinic, 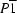

*a* = 8.7240 (5) Å
*b* = 8.9252 (4) Å
*c* = 10.7967 (5) Å
α = 111.312 (2)°
β = 93.084 (3)°
γ = 117.500 (2)°
*V* = 669.24 (6) Å^3^
*Z* = 2
Mo *K*α radiation
μ = 0.11 mm^−1^
*T* = 298 K
0.30 × 0.28 × 0.28 mm

#### Data collection

Bruker SMART CCD diffractometer
Absorption correction: multi-scan (*SADABS*; Sheldrick, 1996 ▶) *T*_min_ = 0.968, *T*_max_ = 0.970
4045 measured reflections
2810 independent reflections
1992 reflections with *I* > 2σ(*I*)
*R*_int_ = 0.013

#### Refinement

*R*[*F* ^2^ > 2σ(*F* ^2^)] = 0.043
*wR*(*F* ^2^) = 0.122
*S* = 1.08
2810 reflections
203 parameters
5 restraints
H atoms treated by a mixture of independent and constrained refinement
Δρ_max_ = 0.15 e Å^−3^
Δρ_min_ = −0.18 e Å^−3^

### 

Data collection: *SMART* (Bruker, 2002 ▶); cell refinement: *SAINT* (Bruker, 2002 ▶); data reduction: *SAINT*; program(s) used to solve structure: *SHELXS97* (Sheldrick, 2008 ▶); program(s) used to refine structure: *SHELXL97* (Sheldrick, 2008 ▶); molecular graphics: *SHELXTL* (Sheldrick, 2008 ▶); software used to prepare material for publication: *SHELXL97*.

## Supplementary Material

Crystal structure: contains datablocks global, I. DOI: 10.1107/S1600536810023949/hb5506sup1.cif

Structure factors: contains datablocks I. DOI: 10.1107/S1600536810023949/hb5506Isup2.hkl

Additional supplementary materials:  crystallographic information; 3D view; checkCIF report

## Figures and Tables

**Table 1:** Hydrogen-bond geometry (Å, °)

*D*—H⋯*A*	*D*—H	H⋯*A*	*D*⋯*A*	*D*—H⋯*A*
N1—H1⋯O1	0.91 (1)	2.14 (2)	2.7257 (17)	121 (2)
N1—H1⋯O2	0.91 (1)	1.88 (2)	2.6366 (17)	139 (2)
O1—H1*A*⋯O2^i^	0.86 (1)	1.72 (1)	2.5675 (16)	165 (2)
O5—H5*A*⋯O3^ii^	0.85 (1)	2.07 (1)	2.907 (2)	169 (2)
O5—H5*B*⋯O3	0.86 (1)	1.95 (1)	2.806 (2)	178 (2)

Symmetry codes: (i) 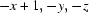; (ii) 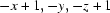.

**Fig. 1.**
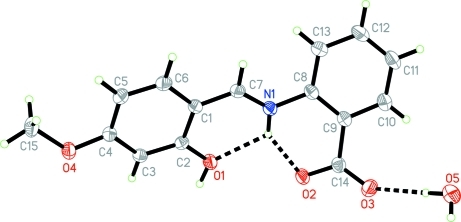
Ellipsoid plot of the title compound at the 30% probability level. Hydrogen atoms are drawn as spheres of arbitrary radius. O–H···N hydrogen bond is drawn by a dashed line.

**Fig. 2.**
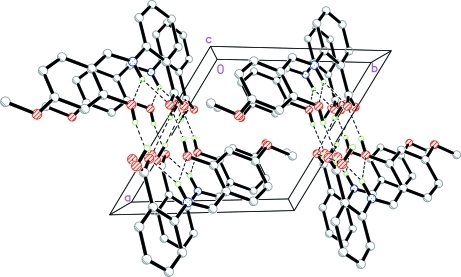
The molecular packing of the title compound, viewed along the c axis. Hydrogen bonds are drawn as dashed lines.

## supplementary crystallographic information

### Comment

The crystal structures of Schiff bases have been widely reported (Alpaslan *et al.*, 2010*a*,*b*; Aritake *et al.*, 2010; Bahron *et al.*, 2010). As a continuation of our work on Schiff bases (Hang, 2010), the present paper reports the title Schiff base compound.

The title compound contains a Schiff base molecule and a water molecule of crystallization (Fig. 1). There exist two intramolecular N–H···O hydrogen bonds in the molecule of the compound. The dihedral angle between the two benzene rings is 25.8 (2)°. The crystal structure is stabilized by intermolecular O–H···O hydrogen bonds (Table 1, Fig. 2).

### Experimental

Equimolar quantities (1 mmol each) of 2-aminobenzoic acid and 4-methoxysalicylaldehyde were mixed and stirred in methanol for 2 h at ambient temperature. The resulting mixture was concentrated under recuced pressure. The residue, purified by washing with cold methanol and diethyl ether, afforded the pure product of the hydrazone compound. Colorless blocks of (I) were obtained by recrystallization of the product from 95% ethanol.

### Refinement

The H atoms attached to N and O atoms were found from a difference Fourier map and refined isotropically, with N–H, O–H, and H···H distances restrained to 0.90 (1), 0.85 (1), and 1.37 (2) Å, respectively. The remaining H atoms were positioned geometrically and refined using a riding model with C–H = 0.93 and 0.96 Å, and *U*_iso_(H) = 1.2 or 1.5*U*_eq_(C).

### Crystal data

**Table:**

C_15_H_13_NO_4_·H_2_O	*Z* = 2
*M**_r_* = 289.28	*F*(000) = 304
Triclinic, *P*1	*D*_x_ = 1.436 Mg m^−^^3^
Hall symbol: -P 1	Mo *K*α radiation, λ = 0.71073 Å
*a* = 8.7240 (5) Å	Cell parameters from 1109 reflections
*b* = 8.9252 (4) Å	θ = 2.6–26.2°
*c* = 10.7967 (5) Å	µ = 0.11 mm^−^^1^
α = 111.312 (2)°	*T* = 298 K
β = 93.084 (3)°	Block, colorless
γ = 117.500 (2)°	0.30 × 0.28 × 0.28 mm
*V* = 669.24 (6) Å^3^	

### Data collection

**Table:**

Bruker SMART CCD diffractometer	2810 independent reflections
Radiation source: fine-focus sealed tube	1992 reflections with *I* > 2σ(*I*)
graphite	*R*_int_ = 0.013
ω scans	θ_max_ = 27.0°, θ_min_ = 2.1°
Absorption correction: multi-scan (*SADABS*; Sheldrick, 1996)	*h* = −6→11
*T*_min_ = 0.968, *T*_max_ = 0.970	*k* = −11→11
4045 measured reflections	*l* = −13→13

### Refinement

**Table:**

Refinement on *F*^2^	Primary atom site location: structure-invariant direct methods
Least-squares matrix: full	Secondary atom site location: difference Fourier map
*R*[*F*^2^ > 2σ(*F*^2^)] = 0.043	Hydrogen site location: inferred from neighbouring sites
*wR*(*F*^2^) = 0.122	H atoms treated by a mixture of independent and constrained refinement
*S* = 1.08	*w* = 1/[σ^2^(*F*_o_^2^) + (0.0595*P*)^2^ + 0.0161*P*] where *P* = (*F*_o_^2^ + 2*F*_c_^2^)/3
2810 reflections	(Δ/σ)_max_ < 0.001
203 parameters	Δρ_max_ = 0.15 e Å^−^^3^
5 restraints	Δρ_min_ = −0.18 e Å^−^^3^

### Special details

**Table:**

Geometry. All e.s.d.'s (except the e.s.d. in the dihedral angle between two l.s. planes) are estimated using the full covariance matrix. The cell e.s.d.'s are taken into account individually in the estimation of e.s.d.'s in distances, angles and torsion angles; correlations between e.s.d.'s in cell parameters are only used when they are defined by crystal symmetry. An approximate (isotropic) treatment of cell e.s.d.'s is used for estimating e.s.d.'s involving l.s. planes.
Refinement. Refinement of *F*^2^ against ALL reflections. The weighted *R*-factor *wR* and goodness of fit *S* are based on *F*^2^, conventional *R*-factors *R* are based on *F*, with *F* set to zero for negative *F*^2^. The threshold expression of *F*^2^ > σ(*F*^2^) is used only for calculating *R*-factors(gt) *etc*. and is not relevant to the choice of reflections for refinement. *R*-factors based on *F*^2^ are statistically about twice as large as those based on *F*, and *R*- factors based on ALL data will be even larger.

### Fractional atomic coordinates and isotropic or equivalent isotropic displacement parameters (Å^2^)

**Table:**

	*x*	*y*	*z*	*U*_iso_*/*U*_eq_	
N1	0.91763 (18)	0.38037 (19)	0.21508 (14)	0.0364 (3)	
O1	0.65275 (15)	0.22394 (15)	−0.01717 (12)	0.0446 (3)	
O2	0.65944 (15)	0.04540 (16)	0.17602 (12)	0.0491 (3)	
O3	0.65792 (17)	−0.01967 (18)	0.35525 (13)	0.0611 (4)	
O4	0.59187 (15)	0.62901 (16)	−0.17000 (12)	0.0459 (3)	
O5	0.68483 (19)	0.0450 (2)	0.63223 (16)	0.0715 (5)	
C1	0.8648 (2)	0.5485 (2)	0.10069 (16)	0.0356 (4)	
C2	0.7092 (2)	0.3956 (2)	−0.00735 (16)	0.0341 (4)	
C3	0.6248 (2)	0.4303 (2)	−0.09650 (16)	0.0364 (4)	
H3	0.5244	0.3306	−0.1682	0.044*	
C4	0.6884 (2)	0.6120 (2)	−0.07994 (16)	0.0371 (4)	
C5	0.8428 (2)	0.7642 (2)	0.02468 (18)	0.0431 (4)	
H5	0.8866	0.8859	0.0346	0.052*	
C6	0.9272 (2)	0.7302 (2)	0.11142 (18)	0.0430 (4)	
H6	1.0301	0.8309	0.1805	0.052*	
C7	0.9597 (2)	0.5313 (2)	0.19950 (17)	0.0377 (4)	
H7	1.0645	0.6404	0.2605	0.045*	
C8	1.0163 (2)	0.3714 (2)	0.31829 (16)	0.0371 (4)	
C9	0.9308 (2)	0.2225 (2)	0.35416 (16)	0.0381 (4)	
C10	1.0311 (2)	0.2180 (3)	0.45585 (19)	0.0486 (5)	
H10	0.9764	0.1205	0.4813	0.058*	
C11	1.2094 (3)	0.3539 (3)	0.5200 (2)	0.0542 (5)	
H11	1.2743	0.3465	0.5865	0.065*	
C12	1.2902 (2)	0.5003 (3)	0.48467 (19)	0.0520 (5)	
H12	1.4098	0.5937	0.5289	0.062*	
C13	1.1959 (2)	0.5100 (2)	0.38442 (18)	0.0449 (4)	
H13	1.2520	0.6092	0.3608	0.054*	
C14	0.7341 (2)	0.0706 (2)	0.29112 (17)	0.0408 (4)	
C15	0.6600 (3)	0.8098 (3)	−0.1699 (2)	0.0545 (5)	
H15A	0.6832	0.9017	−0.0787	0.082*	
H15B	0.5730	0.8035	−0.2330	0.082*	
H15C	0.7693	0.8447	−0.1978	0.082*	
H5B	0.675 (3)	0.022 (3)	0.5468 (12)	0.080*	
H5A	0.591 (2)	0.046 (3)	0.648 (2)	0.080*	
H1	0.8112 (18)	0.2707 (19)	0.166 (2)	0.080*	
H1A	0.5504 (18)	0.145 (3)	−0.0780 (19)	0.080*	

### Atomic displacement parameters (Å^2^)

**Table:**

	*U*^11^	*U*^22^	*U*^33^	*U*^12^	*U*^13^	*U*^23^
N1	0.0349 (8)	0.0350 (8)	0.0348 (7)	0.0161 (6)	0.0043 (6)	0.0143 (6)
O1	0.0436 (7)	0.0320 (6)	0.0458 (7)	0.0131 (5)	−0.0044 (5)	0.0157 (5)
O2	0.0443 (7)	0.0412 (7)	0.0466 (7)	0.0112 (5)	−0.0061 (6)	0.0213 (6)
O3	0.0562 (8)	0.0592 (8)	0.0482 (8)	0.0121 (7)	0.0074 (6)	0.0288 (7)
O4	0.0474 (7)	0.0416 (7)	0.0490 (7)	0.0213 (6)	0.0037 (6)	0.0238 (6)
O5	0.0548 (9)	0.0918 (11)	0.0524 (9)	0.0265 (9)	0.0044 (7)	0.0331 (9)
C1	0.0335 (8)	0.0346 (9)	0.0336 (8)	0.0154 (7)	0.0053 (7)	0.0135 (7)
C2	0.0337 (8)	0.0306 (8)	0.0361 (9)	0.0151 (7)	0.0096 (7)	0.0148 (7)
C3	0.0325 (8)	0.0326 (8)	0.0355 (9)	0.0127 (7)	0.0043 (7)	0.0127 (7)
C4	0.0375 (9)	0.0389 (9)	0.0383 (9)	0.0203 (8)	0.0106 (7)	0.0196 (8)
C5	0.0433 (10)	0.0328 (9)	0.0494 (10)	0.0155 (8)	0.0090 (8)	0.0202 (8)
C6	0.0397 (9)	0.0320 (9)	0.0419 (10)	0.0098 (7)	0.0028 (7)	0.0137 (7)
C7	0.0339 (9)	0.0342 (9)	0.0366 (9)	0.0135 (7)	0.0056 (7)	0.0131 (7)
C8	0.0375 (9)	0.0398 (9)	0.0324 (8)	0.0225 (8)	0.0060 (7)	0.0114 (7)
C9	0.0403 (9)	0.0407 (9)	0.0338 (9)	0.0239 (8)	0.0072 (7)	0.0133 (7)
C10	0.0517 (11)	0.0553 (11)	0.0456 (10)	0.0313 (10)	0.0098 (8)	0.0246 (9)
C11	0.0513 (11)	0.0727 (13)	0.0441 (11)	0.0381 (11)	0.0041 (9)	0.0241 (10)
C12	0.0379 (10)	0.0631 (12)	0.0454 (11)	0.0255 (9)	0.0019 (8)	0.0164 (10)
C13	0.0374 (9)	0.0456 (10)	0.0439 (10)	0.0192 (8)	0.0061 (8)	0.0157 (8)
C14	0.0441 (10)	0.0373 (9)	0.0395 (9)	0.0207 (8)	0.0068 (8)	0.0163 (8)
C15	0.0688 (13)	0.0461 (11)	0.0553 (12)	0.0315 (10)	0.0085 (10)	0.0275 (9)

### Geometric parameters (Å, °)

**Table:**

N1—C7	1.301 (2)	C5—C6	1.360 (2)
N1—C8	1.420 (2)	C5—H5	0.9300
N1—H1	0.912 (9)	C6—H6	0.9300
O1—C2	1.3346 (18)	C7—H7	0.9300
O1—H1A	0.863 (10)	C8—C13	1.393 (2)
O2—C14	1.2625 (19)	C8—C9	1.400 (2)
O3—C14	1.237 (2)	C9—C10	1.391 (2)
O4—C4	1.3474 (18)	C9—C14	1.517 (2)
O4—C15	1.438 (2)	C10—C11	1.379 (3)
O5—H5B	0.858 (9)	C10—H10	0.9300
O5—H5A	0.851 (9)	C11—C12	1.375 (3)
C1—C6	1.408 (2)	C11—H11	0.9300
C1—C7	1.410 (2)	C12—C13	1.377 (2)
C1—C2	1.424 (2)	C12—H12	0.9300
C2—C3	1.384 (2)	C13—H13	0.9300
C3—C4	1.384 (2)	C15—H15A	0.9600
C3—H3	0.9300	C15—H15B	0.9600
C4—C5	1.403 (2)	C15—H15C	0.9600
			
C7—N1—C8	125.26 (14)	C13—C8—C9	120.22 (15)
C7—N1—H1	121.8 (13)	C13—C8—N1	120.48 (15)
C8—N1—H1	112.5 (13)	C9—C8—N1	119.29 (14)
C2—O1—H1A	109.4 (15)	C10—C9—C8	117.87 (16)
C4—O4—C15	118.67 (13)	C10—C9—C14	118.71 (16)
H5B—O5—H5A	105.0 (17)	C8—C9—C14	123.39 (15)
C6—C1—C7	117.43 (14)	C11—C10—C9	121.89 (18)
C6—C1—C2	117.96 (14)	C11—C10—H10	119.1
C7—C1—C2	124.60 (14)	C9—C10—H10	119.1
O1—C2—C3	123.29 (14)	C12—C11—C10	119.34 (17)
O1—C2—C1	117.29 (14)	C12—C11—H11	120.3
C3—C2—C1	119.42 (14)	C10—C11—H11	120.3
C2—C3—C4	120.63 (14)	C11—C12—C13	120.63 (17)
C2—C3—H3	119.7	C11—C12—H12	119.7
C4—C3—H3	119.7	C13—C12—H12	119.7
O4—C4—C3	115.29 (14)	C12—C13—C8	120.03 (17)
O4—C4—C5	123.87 (14)	C12—C13—H13	120.0
C3—C4—C5	120.83 (14)	C8—C13—H13	120.0
C6—C5—C4	118.62 (15)	O3—C14—O2	124.52 (16)
C6—C5—H5	120.7	O3—C14—C9	118.27 (15)
C4—C5—H5	120.7	O2—C14—C9	117.21 (15)
C5—C6—C1	122.51 (15)	O4—C15—H15A	109.5
C5—C6—H6	118.7	O4—C15—H15B	109.5
C1—C6—H6	118.7	H15A—C15—H15B	109.5
N1—C7—C1	127.47 (15)	O4—C15—H15C	109.5
N1—C7—H7	116.3	H15A—C15—H15C	109.5
C1—C7—H7	116.3	H15B—C15—H15C	109.5

### Hydrogen-bond geometry (Å, °)

**Table:**

*D*—H···*A*	*D*—H	H···*A*	*D*···*A*	*D*—H···*A*
N1—H1···O1	0.91 (1)	2.14 (2)	2.7257 (17)	121 (2)
N1—H1···O2	0.91 (1)	1.88 (2)	2.6366 (17)	139 (2)
O1—H1A···O2^i^	0.86 (1)	1.72 (1)	2.5675 (16)	165 (2)
O5—H5A···O3^ii^	0.85 (1)	2.07 (1)	2.907 (2)	169 (2)
O5—H5B···O3	0.86 (1)	1.95 (1)	2.806 (2)	178 (2)

Symmetry codes: (i) −*x*+1, −*y*, −*z*; (ii) −*x*+1, −*y*, −*z*+1.

## References

[bb1] Alpaslan, Y. B., Alpaslan, G., Agar, A. & Isik, S. (2010*b*). *Acta Cryst.* E**66**, o510. 10.1107/S1600536810002989 PMC2983675 21580285

[bb2] Alpaslan, G., Macit, M., Büyükgüngör, O. & Erdönmez, A. (2010*a*). *Acta Cryst.* E**66**, o1178. 10.1107/S1600536810014777 PMC2979202 21579219

[bb3] Aritake, Y., Watanabe, Y. & Akitsu, T. (2010). *Acta Cryst.* E**66**, o749. 10.1107/S1600536810007762 PMC2983984 21580594

[bb4] Bahron, H., Bakar, S. N. A., Kassim, K., Yeap, C. S. & Fun, H.-K. (2010). *Acta Cryst.* E**66**, o883. 10.1107/S1600536810009773 PMC2983940 21580701

[bb5] Bruker (2002). *SAINT* and *SMART* Bruker AXS Inc., Madison, Wisconsin, USA.

[bb6] Hang, Z.-X. (2010). *Acta Cryst.* E**66**, o1650. 10.1107/S1600536810022026 PMC3006874 21587878

[bb7] Sheldrick, G. M. (1996). *SADABS* University of Göttingen, Germany.

[bb8] Sheldrick, G. M. (2008). *Acta Cryst.* A**64**, 112–122. 10.1107/S0108767307043930 18156677

